# Littoral cell angioma of the spleen in a patient with previous pulmonary sarcoidosis: a TNF-α related pathogenesis?

**DOI:** 10.1186/1477-7819-9-106

**Published:** 2011-09-19

**Authors:** Stefanie Cordesmeyer, Manfred Pützler, Ulf Titze, Harald Paulus, Matthias W Hoffmann

**Affiliations:** 1 Department of Transplantation Medicine, University Hospital, Albert-Schweitzer Campus 1, 48149 Münster, Germany; 2 Department of General and Visceral Surgery, Raphaelsklinik, Loerstraße 23, 48143 Münster, Germany; 3 Department of Radiology, Raphaelsklinik, Loerstraße 23, 48143 Münster, Germany; 4 Department of Pathology, University Hospital, Albert-Schweitzer Campus 1, 48149 Münster, Germany; 5 Internal Medicine, Private Practice, Himmelreichallee 37, 48149 Münster, Germany

**Keywords:** Splenic tumor, littoral cell angioma, visceral organ malignancies, sarcoidosis, TNF-α

## Abstract

**Background:**

Littoral cell angioma (LCA) is a rare vascular tumor of the spleen. Generally thought to be benign, additional cases of LCA with malignant features have been described. Thus, its malignant potential seems to vary and must be considered uncertain. The etiology remains unclear, but an immune dysregulation for the apparent association with malignancies of visceral organs or immune-mediated diseases has been proposed.

**Case Presentation:**

We report a case of LCA in a 43-year old male patient who presented with a loss of appetite and intermittent upper abdominal pain. Computed tomography showed multiple hypoattenuating splenic lesions which were hyperechogenic on abdominal ultrasound. Lymphoma was presumed and splenectomy was performed. Pathological evaluation revealed LCA.

**Conclusions:**

LCA is a rare, primary vascular neoplasm of the spleen that might etiologically be associated with immune dysregulation. In addition, it shows a striking association with synchronous or prior malignancies. With about one-third of the reported cases to date being co-existent with malignancies of visceral organs or immune-mediated diseases, this advocates for close follow-ups in all patients diagnosed with LCA. To our knowledge, this report is the first one of LCA associated with previous pulmonary sarcoidosis and hypothesizes a TNF-α related pathogenesis of this splenic tumor.

## Background

Vascular tumors are the most common primary neoplasms of the spleen. Among these, LCA is a very rare tumor which arises from the littoral cells lining the sinuses of the splenic red pulp. The tumor displays a unique immunohistochemical phenotype of dual endothelial and histiocytic differentiation but is difficult to differentiate from other benign and malignant splenic lesions preoperatively. Thus, diagnosis is usually established after elective splenectomy. Currently, both etiology as well as biological behavior remain uncertain. Increasing numbers of LCA in association with autoimmune disorders or visceral organ tumors have been reported which hypothesizes an immunological association of this entity.

## Case presentation

**Figure 1:**
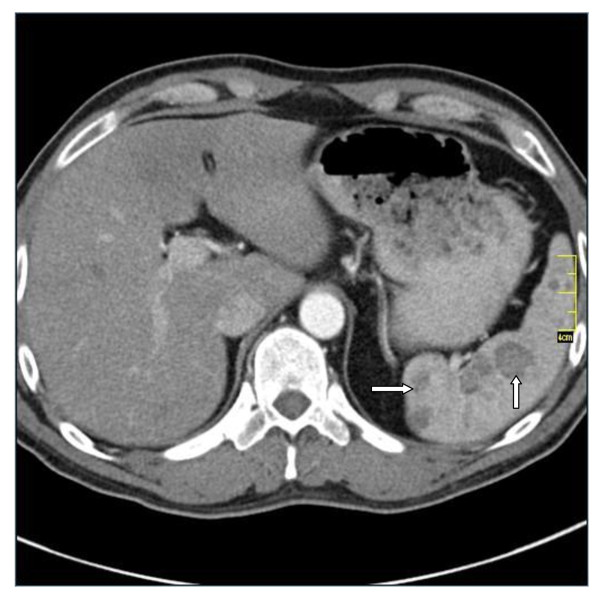
**Computed tomography showing multiple hypoattenuating lesions (arrows)**.

A 43-year old male presented with non-specific clinical symptoms such as loss of appetite and intermittent upper abdominal pain which improved slightly with antacid medication. His medical history was unremarkable except for pulmonal sarcoidosis in his twenties which had been treated with corticosteroid medication. Both physical examination and laboratory tests were without pathological findings. An ulcerous lesion in the duodenum was detected gastroscopically and computed tomography was performed to exclude an external compressing tumor. CT scans (Figure 1) did not detect any tumor but revealed mild splenomegaly with multiple hypoattenuating nodules with a maximum diameter of 2.5 cm which were contrast-enhancing in the late portal venous phase.

**Figure 2:**
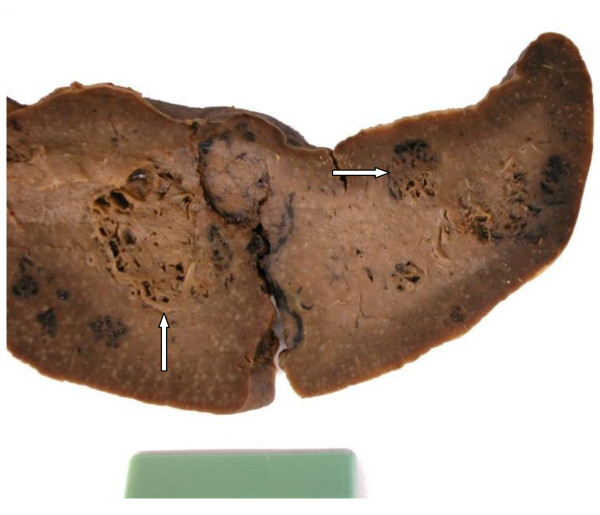
**Splenectomy-specimen revealing multiple nodular lesions with spongy appearance (arrows)**.

Abdominal ultrasound revealed multiple hyperechogenic splenic lesions without evidence of metastatic disease or infectious origin and hemangioma was assumed. Despite the absence of adenopathy, lymphoma was finally considered the most likely diagnosis, given the quantity of the nodules as well as their distribution within the spleen and splenectomy was advocated. After appropriate preoperative vaccination for *Streptococcus pneumoniae, Haemophilus influenca B *and *Neisseria meningitidis*, laparoscopic splenectomy was performed. The cut surface of the 12 × 8 × 4 cm specimen (Figure 2) showed multiple nodular lesions with spongy appearance, varying from 0.5 to 2 cm in greatest dimension.

**Figure 3:**
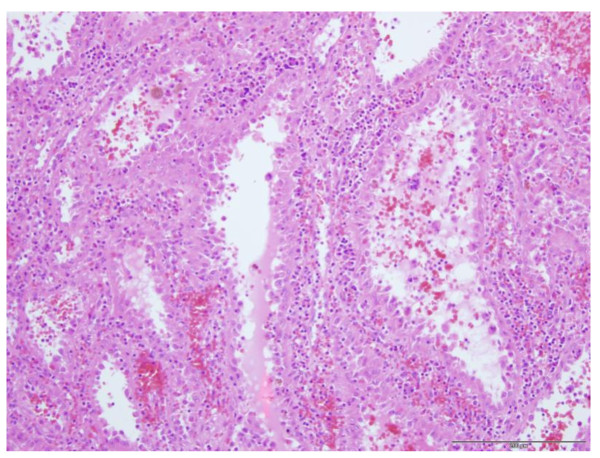
Neoplastic sinuses lined by a single cell layer (20× obj, HE-staining).

**Figure 4:**
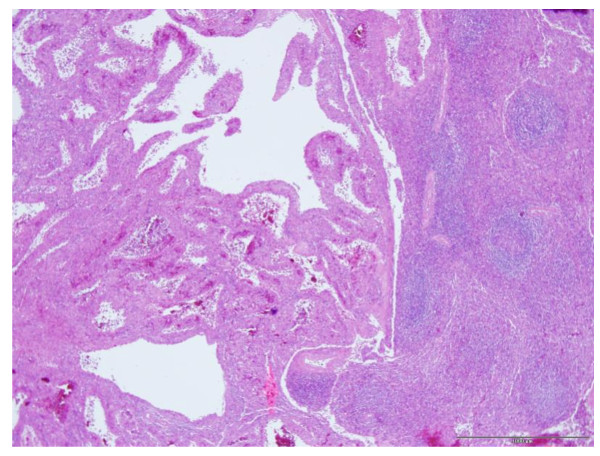
**Regular splenic parenchyma (right) and tumor (left) composed of lacunae filled with oedematous fluid and blood (4× obj, HE-staining)**.

**Figure 5:**
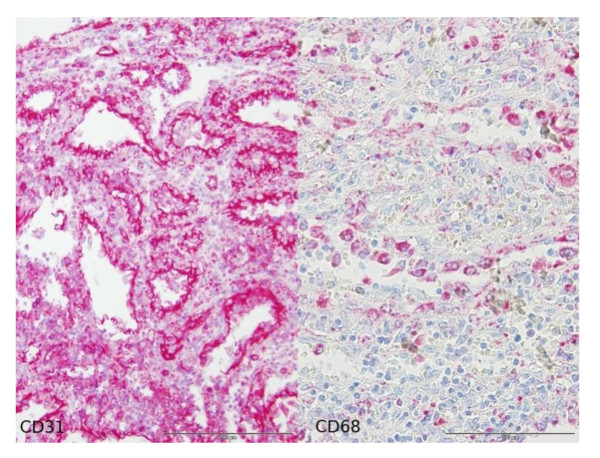
**Combined expression of endothelial (CD31) and histiocytic (CD68) markers in immunohistochemical staining**.

Microscopically, these lesions were composed of cavernous sinuses which were lined by a single layer of tall cells (Figure 3) lacking typical endothelial features. The lacunae were filled with edematous fluid and blood (Figure 4). Immunohistochemical staining (Figure 5) was positive for both endothelial (CD 31) and histiocytic (CD 68) markers. No cytologic atypia and mitotic figures were found.

The combination of morphological and immunohistochemical analysis presenting this hybrid endothelial-histiocytic phenotype established the diagnosis of LCA. The postoperative course was uneventful and the patient was discharged on day five. He will be followed-up closely for the occurrence t of visceral neoplasms.

## Discussion

Littoral cell angioma (LCA) is a rare vascular tumor that occurs exclusively in splenic tissue and was first described by Falk et al in 1991 [1]. It originates from the specialized endothelial cells lining the sinus channels of the splenic red pulp, called "littoral cells". LCA show neither gender nor age predilection. It might be discovered incidentally in completely asymptomatic patients or in those presenting with non-specific clinical symptoms like in our case. About 50% of all patients show splenomegaly or signs of hypersplenism like anemia or thrombocytopenia [1]. On ultrasound, the findings vary widely from heterogeneous echotexture without specific nodules [2] to hyperechogenic [3], hypoechogenic [4] or isoechogenic [5] appearing lesions [6-8]. Computed tomography typically shows multiple hypoattenuating nodules [9]. These findings are non-specific and several differential diagnosis have to be considered. These include benign neoplasms like hamartoma or hemangioma but also metastatic diseases or disseminated infections [2]. Since our patient did not present with adenopathy or disease in other organs, metastatic disease was considered an unlikely diagnosis. Considering disseminated infections, we had to exclude fungal disease, septic emboli and granulomatous diseases such as sarcoidosis and tuberculosis. Associated adenopathic, pulmonary and mediastinal diseases suspecting sarcoidosis or tuberculosis were not detected. Mycobacterium avium-intracellulare complex, Pneumocystic carinii and disseminated Kaposi sarcoma may also cause splenic masses but are typically seen in immunocompromised individuals. After elaborating for these differentials, a definite diagnosis is often still difficult to obtain and splenectomy is subsequently performed for further evaluation. Gross pathology typically shows multiple focal blood-filled nodules and microscopic examination reveals anastomosing vascular channels lined with tall endothelial cells and papillary fronds [1,7,9].

Since the littoral cells have features intermediate between those of endothelial cells and macrophages, they show a hybrid endothelial-histiocytic phenotype on immunohistochemical staining. Expression of endothelial marker factor VIII-related antigen and also of histiocytic markers such as CD68 and lysozyme is thought to be characteristic for LCA [1,6,7] and establishes the final diagnosis.

Generally thought to be benign, there are additional reports of LCA with malignant features which were divided into a low-grade variant (littoral cell hemangioendothelioma [10]) and the tumor's malignant counterpart, littoral cell angiosarcoma [11]. Therefore, its biological behavior seems to vary and must be considered uncertain [9].

The etiology of this neoplasm also remains unclear, but for its apparent association with visceral organ tumors and immune-mediated diseases in one-third of the reported cases to date [12-16], an etiological association with immune dysregulation has been proposed [1,17,18]. To support this contention, there are more reports of LCA in patients with long-term immunosupressive therapy, i.e. after renal transplantation [18] or for systemic lupus erythematosus [19]. Reviewing literature for similarities of immune-mediated diseases and LCA we found two cases of LCA in patients with Gaucher's disease [20,21], a lipid storage disorder characterized by accumulation of cerebroside in the cytoplasm of macrophages due to deficiency of an enzyme, glucocerebrosidase [20,21]. Both LCA and Gaucher's disease involve lysozymes. Since the likelihood of chance occurrence of two rare disorders in one patient is low, Gupka et al suggested a possible pathophysiologic association [20].

Since our patient had a history of pulmonal sarcoidosis, we concentrated on possible immunological links between these two entities.

In sarcoidosis, a multisystemic granulomatous disorder of unknown origin, the inflammatory response is characterized by the increased production of several pro-inflammatory cytokines which mainly belong to the tumor necrosis factor family. Tumor necrosis factor-alpha (TNF-α) is considered the pivotal factor in the formation of granulomas by mediating inflammation and cellular immune response among the cytokines involved [22]. TNF-α is released by macrophages and binds to two types of receptors, the 55 kDa (TNF receptor I: TNF RI) and the 75 kDa receptor (TNF RII) [23]. Elevated serum levels of these receptors have been demonstrated in a variety of diseases, e.g. rheumatic diseases, malignancies and Crohn's disease, and are thought to reflect the disease activity. Since LCA is a neoplasm arising from the lining cells, the spleen's macrophages, this could also be an area of increased production of TNF-α, eventually contributing to the pathogenesis of LCA, since inflammatory cells including TNF-α are known to have powerful effects on tumor development, producing an attractive environment for tumor growth by facilitating genomic instability and promoting angiogenesis. The inflammatory cells as well as the chemokines and cytokines they produce finally influence the whole tumor organ, regulating the growth, migration and differentiation of all cell types in the tumor microenvironment, including neoplastic cells, fibroblasts and endothelial cells [24]. Thus, a TNF-α related pathogenesis of LCA could also provide an explanation for both the occurrence of synchronous or metachronous visceral organ tumors as well as the affection for immune-mediated diseases.

## Conclusions

LCA is a rare, primary vascular neoplasm of the spleen. Currently, both etiology and biological behavior remain unclear, but an underlying immune dysregulation has been proposed for LCA's association with malignancies of visceral organs or immune-mediated disorders in about one-third of the reported cases. Our case presentation supports the assumption of an association of LCA and an altered immune status, hypothesizing a TNF-α-related pathogenesis of this splenic tumor. Close follow-ups of patients diagnosed with LCA for subsequent development of additional tumors is mandatory.

## Consent

Written informed consent was obtained from the patient for publication of this case report and any accompanying images. A copy of the written consent is available for review by the Editor-in-Chief of this journal.

## Competing interests

The authors declare that they have no competing interests.

## Authors' contributions

SC reviewed relevant literature and wrote the initial draft. MP reviewed the draft and contributed the CT scans. UT contributed the histological images. HP provided clinical expertise and reviewed the manuscript. MWH performed the surgery and reviewed the manuscript. All authors read and approved the final manuscript.
